# Adaptation to drought in arid and semi-arid environments: Case of the Zambezi Valley, Zimbabwe

**DOI:** 10.4102/jamba.v7i1.144

**Published:** 2015-03-11

**Authors:** Emmanuel Mavhura, Desmond Manatsa, Terence Mushore

**Affiliations:** 1 Department of Geography, Bindura University of Science Education, Zimbabwe

## Abstract

Small-scale rain-fed agriculture is the main livelihood in arid to semi-arid regions of sub-Saharan Africa. The area is characterised by erratic rainfall and frequent droughts, making the capacity for coping with temporal water shortages essential for smallholder farmers. Focusing on the Zambezi Valley, Zimbabwe, this study investigates the impact of drought on food security and the strategies used by smallholder farmers to cope with drought. We used meteorological data and interviews to examine the rainfall variability in the study area and the drought-coping mechanisms employed by smallholder famers respectively. The results show that there are various strategies used by smallholder farmers to cope with the impact of drought. These strategies include drought-tolerant crop production, crop variety diversification, purchasing cereals through asset sales, non-governmental organisations’ food aid and gathering wild fruit. However, consecutive droughts have resulted in high food insecurity and depletion of household assets during droughts. Smallholder farmers in the valley have also resorted to a number of measures taken before, during and after the drought. Still, these strategies are not robust enough to cope with this uncertainty.

## Introduction

Attaining Millennium Development Goals (MDGs) on eradicating extreme poverty and hunger remains a challenge in areas of sub-Saharan Africa (SSA) (Enfors & Gordon 2008; Nyakudya & Stroosnijder 2011). For example, in 2012 it was estimated that 72% of the Zimbabwean population were living below the national poverty datum line and that 11.5% of the population were in severe poverty (United Nations Development Programme [UNDP] 2013a, 2013b). Drought is leading to extreme hunger in SSA. About 60% of the population in this region is vulnerable to drought, whilst 30% is regarded as highly vulnerable (Ngaka 2012). The Zambezi Valley in Zimbabwe is no exception, as it is estimated that about 65% of the country receives less than 500 mm of rain per year (Nyakudya & Stroosnijder 2011). This implies that most of the farming in the country takes place under arid and semi-arid conditions. Despite this status quo, a greater concern for the farming community is the projected broad reduction of about 5% – 10% in the annual rainfall nationwide (Nyakudya & Stroosnijder 2011).

Small-scale rain-fed agriculture is the main livelihood source in arid and semi-arid areas of SSA. The yield levels in such farming systems are very low especially during years of severe drought. In some cases a little surplus is realised which is then saved for other household needs. In response to the low yields, smallholder farmers diversify their sources of income. The diversification is also a way to accumulate wealth. However, the security of the livelihoods of smallholder farmers in this environment remains closely linked with the productivity levels of the local agro-ecological zones, which are hindered to a large extent by water availability (Millennium Ecosystem Assessment [MEA] 2005; Stringer et al. 2009). The arid and semi-arid areas are characterised by high atmospheric evaporation and highly variable spatial and temporal precipitation that makes rain-fed farming a risk economic activity (Nyakudya & Stroosnijder 2011). The erratic rainfall and frequent droughts make the capacity for coping with temporal water shortages important for smallholder farmers (Enfors & Gordon 2008; Stringer et al. 2009).

This study investigates the impact of drought on food security and the strategies amongst smallholder farmers in the Zambezi Valley, Zimbabwe to cope with drought. A case study approach is adopted in which rural communities are acknowledged as people taking a leading role in the creation, facilitation and enacting of drought adaptation strategies. This place-specific approach provides insight into what is occurring in the study area. It also allows for holistic consideration of the drought issue, its local manifestation and how the local experiences may help other rural communities in their adaptation to drought (Kiem & Austin 2013). The findings are discussed in relation to aspects of disaster risk reduction amidst climate variability and drought.

### Drought hazard

Smallholder farmers in arid to semi-arid SSA often experience insufficient food as a result of water stress (Nyakudya & Stroosnijder 2011). Water for crop production and poverty in arid and semi-arid regions of Zimbabwe are strongly linked. The main challenge to food security for many communal and small-scale commercial farmers in the country is water for crop production. Unlike the common view, the shortage of water has more to do with precipitation variability and with large unproductive flows in the field water balance, than with the total amount of rainfall (Enfors & Gordon 2008). The distinction between meteorological and agricultural drought is therefore important here. A meteorological drought means that cumulative precipitation for the entire growing season is less than the amount required to produce a crop (Smith & Petley 2009). This situation normally results in total crop failure. Agricultural drought, however, could happen during seasons with higher precipitation totals than those defined as meteorological droughts. It happens when the cumulative soil moisture available for plants is greatly lower than the cumulative water requirements for crops (Nyakudya & Stroosnijder 2011). Although the cumulative precipitation may be enough to produce a crop, there is a shortage of moisture available for plants, resulting in yield reduction or total failure of crops (Enfors & Gordon 2008).

Agricultural drought is much more common than meteorological drought. This is caused by a variety of factors, such as dry spells, water losses from the field via run-off, drainage of soils and evaporation rates (Enfors & Gordon 2008). Dry spells occur as short periods of water stress that last for a few weeks during crop growth (Nyakudya & Stroosnijder 2011). At the same time crops may experience water stress earlier than needed, even though there is enough rainfall required by crops. This phenomenon is called induced drought. A period of water stress ranging from 5 to 15 days is viewed as harmful in SSA (Nyakudya & Stroosnijder 2011). Enfors and Gordon (2008) show that reduction in crop yields caused by intra-seasonal dry spells occurs every three out of four seasons in semi-arid agricultural systems. The inadequate water availability for crops and a wide range of other constraints make the small-scale agricultural system a highly uncertain food and income source in these areas (Enfors & Gordon 2008).

The characteristics of drought are varied. They include stress within the environment, deterioration in the vegetation cover, losses in agricultural production, loss of arable land, soil erosion and increased stress on the economy, amongst others (Nyakudya & Stroosnijder 2011). Zimbabwe experienced this scenario during the El Niño years, such as the 1991/1992 season. Irrigation becomes a viable strategy for crop production in such drought-prone environments. However, exploring this strategy in the near future is a challenge in Zimbabwe because of the high cost of irrigation infrastructural development and the limited suitable hydrogeological conditions for irrigation development. Other strategies of mitigating agricultural drought include the adoption of crop management practices such as planting drought-tolerant crops, using short season cultivars, rainwater harvesting (RWH) techniques, soil moisture retention techniques and conservation farming (Nyakudya & Stroosnijder 2011). RWH techniques mitigate the risks associated with intra-seasonal dry spells, thereby bridging the gap between rainfall events, whilst conservation tillage enables improved timing of farming operations. Use of drought-tolerant crops such as pearl millet (*Pennisetum typhoides, Pennisetum Americana* or *Pennisetum glaucum)* and sorghum (*Sorghum bicor*), with minimum water requirements of 300 mm or less and 400 mm respectively, are a better option in semi-arid areas. Pearl millet has an added advantage of giving economic yields in highly degraded soils that cannot support cereals.

### Site description: Zambezi Valley

**FIGURE 1:**
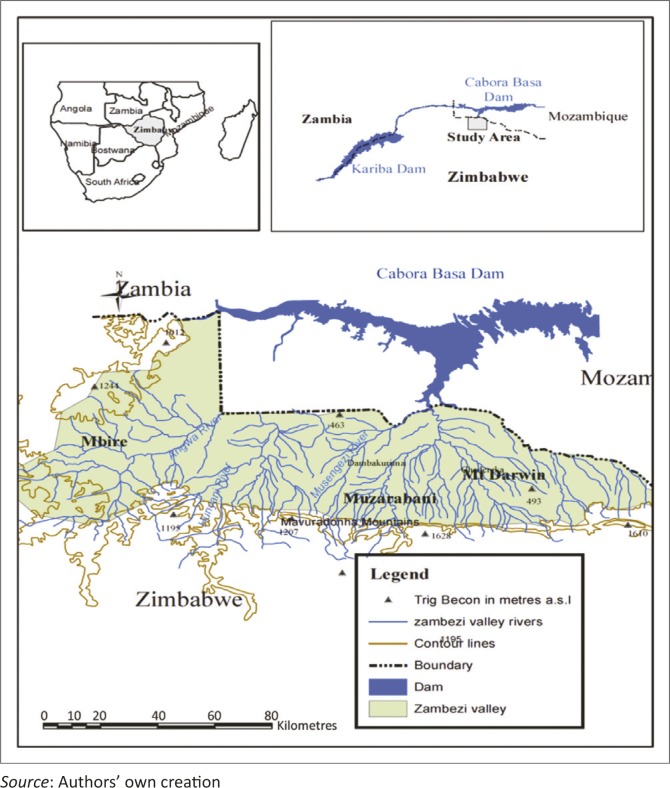
Map of the Zambezi Valley, Zimbabwe.

This case study focused on smallholder farmers in the Zambezi Valley, in the Mashonaland Central Province of Zimbabwe (Figure 1). The valley is shared by three districts: Mbire, Muzarabani and Mt Darwin. About 250 000 people live in the valley (ZimStats 2012). Their main livelihoods are smallholder farming and livestock production. Smallholder farmers have a mean land holding size of about 1.1 ha per household. They grow maize, pearl millet, sorghum and rapoko (*eleusine coracana* or *zviyo* in Shona, the local language) for subsistence, and cash crops such as cotton and tobacco. However, the main cereal crop is maize (*Zea mays L.*). Although there are other cereals which are not very sensitive to water stress, smallholder farmers prefer maize because it forms the staple food in the area (Nyakudya & Stroosnijder 2011). There are also other non-farming income sources that play an essential role in the local economy.

The Zambezi Valley is a semi-arid to arid region located in agro-ecological region IV. The region is characterised by low annual precipitation of 450 mm – 650 mm, seasonal droughts and severe intra-season dry spells. The rain season is unimodal, extending from mid-November up to the end of March (hot, wet season). The climate of the Zambezi Valley is largely controlled by global atmospheric circulation patterns, chief amongst them the movement of the inter-tropical convergence zone (ITCZ) that determines the annual seasonality of precipitation across tropical Africa (Mavhura et al. 2013; Stringer et al. 2009). Because of high temperatures during the summer season convectional rainfall is experienced at times. However, the valley is characterised by extreme variations in rainfall both spatially and temporarily (Madamombe 2004). Year to year droughts are projected to increase across southern Africa (Kiem & Austin 2013).

## Methods and materials

The materials used in this study could be divided into two types: meteorological data and semi-structured interviews.

### Seasonal rainfall variability over the Zambezi Valley

**FIGURE 2:**
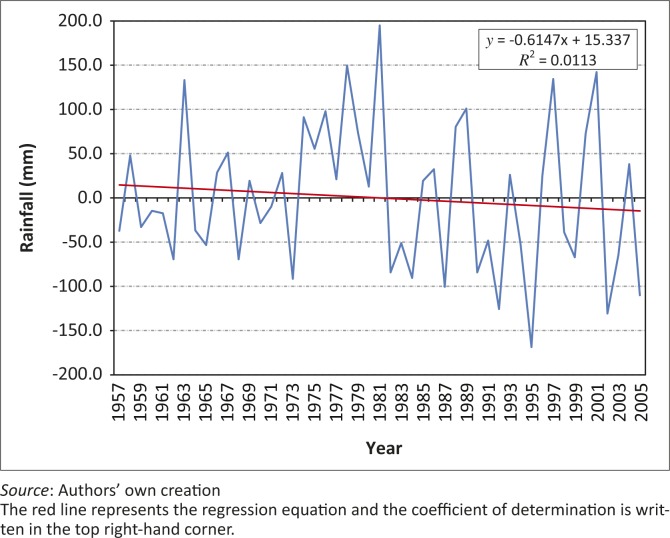
Temporal manifestation of the rainfall anomalies for the Zambezi Valley for the period 1957–2005.

Firstly, the meteorological data consists of monthly rainfall data for Kanyemba Station in the Zambezi Valley collected from the Meteorological Services Department (MSD), Zimbabwe. The data ranges from 1956 to 2005 (Figure 2). The anomalies are derived from the mean constituting all the whole data for that period.

The rainfall in the Zambezi Valley is highly seasonal (90% occurring between November and March), often with a mid-season dry spell that occurs during critical periods of crop growth. Precipitation typically occurs on a number of isolated days and locations, seldom exceeding 50 rain days per annum. The rainfall varies significantly between months and seasons, with the months comprising the season having received rainfall total ranging from 0 mm to 21 mm and the season with the lowest rainfall having received 51 mm (Table 1). Some months from December to March have received rainfall in excess of the mean seasonal total. The high variability of rainfall makes the area more susceptible to drought. The onset and cessation dates also vary widely. The mean onset and cessation dates are around 22–25 November and 09–13 March respectively. The onset and cessation dates can vary by as much as 23 and 27 days respectively, suggesting that the onset and cessation dates are barely reliable. The relatively short season ensures that the rainfall averaging small amounts of 219.3 mm, which comes to constitute the season, is spread over a very limited space of time. However, linear regression analysis for the seasonal total demonstrates that although the trend is negative, it is statistically insignificant, suggesting that the rainfall total has remained relatively constant for much of the period. These rainfall characteristics limit crop production for the region.

**TABLE 1:** Monthly seasonal rainfall total characteristics for the Zambezi Valley.

Characteristics	Oct.	Nov.	Dec.	Jan.	Feb.	Mar.	Oct., Nov., Dec.	Jan., Feb., Mar.	Season
Mean (in mm)	12.5	55.9	176.6	176.6	180.0	92.0	73.7	145.5	219.3
Maximum (in mm)	89.8	199.2	455.7	455.7	585.4	422.1	202.3	322.8	414.4
Minimum (in mm)	0.0	1.3	21.2	21.2	13.9	0.0	20.0	29.1	50.7
Range (in mm)	89.8	197.9	434.5	434.5	571.5	422.1	182.4	293.7	363.7

### Interviews

Secondly, data was collected through semi-structured interviews. Farmers from 60 different households were interviewed between May and December 2013. The duration of the interviews ranged between 60 and 90 min. The respondents were villagers who were either household heads or household breadwinners. They were chosen in agreement with the traditional leadership and the ward councillors. The criteria used ensured that the sampled respondents were from different sizes of households, income and location. The criteria reduced the risk of sampling bias towards either households earning a high income, or other social groups who enjoyed high status with the community, which would likely influence the results on community's coping capacity. Their ages ranged from 25 to 65 years.

The aim of the interviews was to investigate the impact of drought on food security and the strategies employed by smallholder farmers in the valley to cope with the condition. The interviews focused on three main themes (Table 2). The interview included a ranking exercise. The respondents ranked their current food and income sources according to importance. This approach fuelled the discussion and yielded a quantitative approximation of the relative importance of their food and earnings. This shed further light on the strategies employed by households to deal with drought and a more nuanced view of their coping capacity. The approach also served as a method of triangulating the interviews. However, we did not focus on all crops when considering the community's agricultural adaptations. Instead, we considered only maize because it is the staple food in the community. The impact of drought on maize crop is likely to have the most adverse impact on the wellbeing of the community.

**TABLE 2:** Main interview themes.

Theme number	Description
1	Impact of drought on food security
2	Strategies to deal with drought-induced food shortages
3	Drought adaptations from the insight of Disaster Risk Reduction

The qualitative data were grouped and analysed thematically (Enfors & Gordon 2008). When the data were structured into themes, this yielded practical analytical categories that simplified the exploration of common responses and ways of reasoning regarding key issues. The structuring also made it possible to identify statements that were diverging from such patterns. The qualitative responses were coded into a set of defining variables. For example, the variable on food shortage included responses to questions regarding the household's current food status, the number of meals consumed on daily basis, and the variety of food eaten. This variable was analysed as high or low. In addition to this all defining variables were cross-checked against each other as a way of searching for potential trends in the material. Another layer of analysis was added by grouping the defining variables into a few broader variables (Table 3). For example, the food security category was evaluated in terms of both food shortages and household food production. The choice of these defining variables was largely influenced by the fact that rain-fed agriculture is the major source of livelihood in the Zambezi Valley.

**TABLE 3:** Categories and variables used in the analysis of interview data.

Category	Defining variable	Discussion question or topic
Food security	Food shortage	Based on questions regarding perception on current household food situation, number of meals per day and types of food eaten
	Household food production	Based on ranking exercise. Classified as high if >50% of the household food needs is produced within the own system, incl. current harvest of staple and cash crops, food stored from previous harvest, and poultry
Dealing with food shortages	Food relief	Based on questions regarding relief from NGOs, government
	Non-agro-based foods	Based on questions regarding other sources of income to cushion famers
	Seasonal harvest	Based on questions regarding local food production and markets
DRR adaptations to drought	Before drought	Based on questions regarding activities in anticipation of drought
	During drought	Based on questions regarding options for limiting drought
	After drought	Based on questions regarding options for recovering from drought

DRR, Disaster Risk Reduction; NGOs, non-governmental organisations.

## Results

### Impact of drought on food security

The interviews revealed that high food insecurity in the Zambezi Valley was the major concern amongst smallholder farmers in the area. This was a result of the persistent droughts experienced over the decades. Of all the respondents, only 5 households (*n* = 60) had harvested enough cereals for their home consumption at the end of the 2011/2012 farming season. More than 85% of the interviewees stated that they were experiencing food shortages during harvest, whilst 25% stated that their harvests could last them 3 months only. As a result the food insecurity levels varied from household to household. Some households had to reduce the amount of food per meal and/or number of meals eaten on a daily basis, whilst others had to change their diets. The poor harvest in the valley resulted in maize price increases from $12 to $60 per 50 kg bag between May and December 2012. At the same time prices for their livestock (cattle, goats and sheep) dropped by over 15%. The dramatic decrease in price was partly caused by market competition and oversupply of livestock at the market when smallholder farmers were looking for money to outsource food. At the same time, the drought had reduced the fodder and grazing pastures to an extent that the livestock lost weight, leading to poor health.

### Strategies to cope with food shortages

**FIGURE 3:**
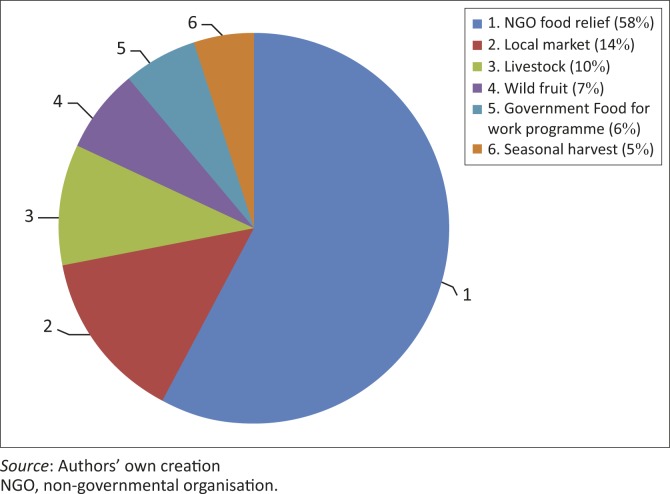
Strategies used by households to source food after a drought.

The ranking exercise revealed that during the 2011/2012 season only 15% of the food needed by the households in the Zambezi Valley was produced within the community's own agricultural systems. Drought had induced food insecurity in the valley. To cover the shortfall, smallholder farmers used a number of strategies (Figure 3). The most common strategy was relying on non-governmental organisation (NGO) food aid (58%), followed by buying food on the local market (14%); the least common was obtaining food from the seasonal harvest (5%). Some households depended on government food for work programmes (6%) and gathering wild fruit (7%).

Household food expenditure increased because smallholder famers had to sell their livestock at relatively low prices whilst buying food at high prices. This needed other sources of income to cover the rising costs. Production of cash crops (cotton and tobacco) and small-scale horticultural activities were the main sources of income for the smallholder farmers (Table 4). Unfortunately, these activities were severely affected by the consecutive droughts in the area. Therefore their total share (32%) of the average income contribution after a drought was not very significant. This made the community increasingly reliant on non-agro-ecosystem-based income sources, such as informal trading and sale of household goods. However, these were not viable sources of income because of the ripple effects of drought. Interestingly, the diversification towards less rainfall-dependent sources of income was viewed as the norm for smallholder farmers in many areas (Enfors & Gordon 2008). The results of this study show that 67% of income was generated from such sources. These included livestock sales, informal trading, household property sales, savings and formal employment.

**TABLE 4:** Sources of household income after drought.

Source	Households that use income source† (%)	Average income contribution from source (%)
Livestock sales	40	35
Cash crops (cotton, tobacco)	30	25
Informal trading	28	22
Small-scale horticultural activities	17	8
Formal employment	7	5
Sale of household goods	5	3
Savings	3	2

† Most households used a number of different income sources, explaining why the total exceeds 100%.

To cope with the persistent droughts, 3% of the interviewed households were forced to fall back on savings, whilst 5% sold household goods that were accumulated over the preceding seasons. Had they accumulated much, the contribution of the two could have increased significantly as alternative sources of income to avert hunger and starvation. However, the average income contribution of savings and sale of household goods remained low because of extreme poverty. At the same time, resorting to savings and sale of asset holdings prevented many of the smallholder farmers to make large farm investments which could have reduced the impact of droughts. For example, one farmer who had acquired various farming equipment was compelled to sell them so as to purchase food. Therefore, the family food needs of that farmer were provisionally met, at the expense of improved tillage equipment. This meant that the family would be unable to produce food in the coming season even if there were normal to above normal rainfall. Comments similar to the ones below were made by a quarter of the interviewees, showing that the depletion of asset holdings which were induced by drought had negatively impacted on their welfare:

- ‘I was forced to sell two beasts so that I could afford to buy food which can last my family until the next season.’
- ‘All of our income goes to food now, and therefore we can't afford paying tuition for our children.’
- ‘We no longer have anything to dispose of so as to buy food. We experience droughts every year.’

### Drought adaptations from the perspective of Disaster Risk Reduction

In their adaptation to recurrent seasonal droughts, smallholder farmers in the valley have resorted to a number of measures taken in different periods of drought. Their activities resemble the disaster management cycle in which activities and measures are taken up before, during and after a disaster with the aim of avoiding the disaster, reducing its impact or recovering quickly from its losses (Caymaz, Volkan & Erenel 2013; Smith & Petley 2009). These measures have different temporal characteristics, and we analyse them in three phases, each one with its own objectives.

#### Before drought disaster

The measures taken by the smallholder farmers before a drought were aimed at avoiding the drought risks. The drought prevention measures included the tradition of holding rituals (*mukwerera* in Shona, the local language) before the rain season, the use of traditional drought warning and forecasting systems, and monitoring the number of grazing animals. For example, smallholder farmers understood that the production of a large number of wild fruit and a large population of insects just before the beginning of the rainy season signal an impending drought. Other measures taken by local farmers had to do with the choice of drought-resistant crops, planting short season cultivars, using zero tillage, stockpiling supplies and staggering the planting time of fields so as to minimise risk of entire crop failure.

The tradition of holding rituals before the rainy season was meant to ask their ancestors and God for abundant rainfall so that they could harvest enough for their consumption. For this to happen, several rules and regulations have been established and recognised by all smallholder farmers. The rules explain the planting decisions, when to refrain from working in the fields, and how to appease their ancestors in order to guarantee a large enough harvest. Smallholder farmers informed us that they respected their customs and each year they hold ceremonies to worship their ancestors before the harvest.

**FIGURE 4:**
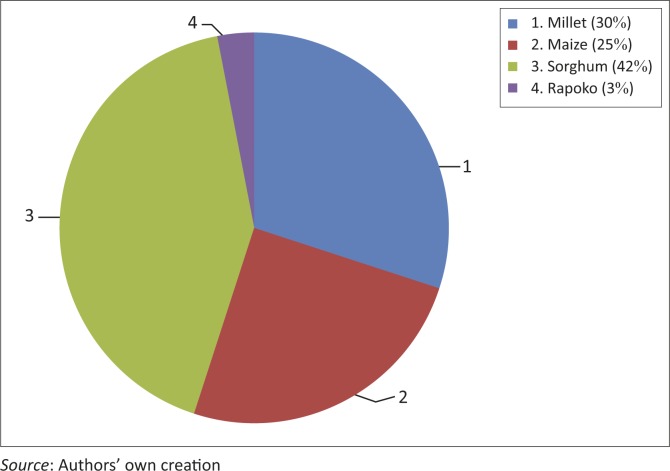
Proportion of households who grow drought-tolerant cultivars and maize.

Results from interviews showed that (98%) of households grew drought-tolerant cultivars such as pearl millet, sorghum, rapoko and cotton. In response to drought impacts, crop fields were planted at different times to reduce risk of entire crop failure. Cropped area varied greatly but almost every household had a piece of land under a drought-tolerant crop. Those with small land holdings of about an acre or less indicated preference of growing maize at the expense of drought-tolerant crops and were therefore more vulnerable in the event of a drought. The highest proportion (42%) of the households grew sorghum; 30% pearl millet; 25% maize varieties and 3% rapoko (Figure 4). Interviews revealed that the households which grew drought-resistant crops obtained higher yields as compared to those which grew maize.

#### During drought disaster

Measures taken during periods of drought were aimed at lessening or limiting the severity of the adverse impacts of the drought, i.e. mitigation. The survey revealed that smallholder farmers used water conservation techniques such as RWH, *in situ* water harvesting, run-off water harvesting and flood diversion in order to improve their crop harvest. They also put some of their livestock on sale, and engaged in food for work programmes.

#### After drought disaster

The smallholder farmers took measures to transfer risks. These initiatives were employed in response to a drought event with the aim of achieving early recovery and rehabilitation of affected households. In order to improve the capacity to resist and recover quickly from the drought impact, smallholder famers made sure that their income from non-agricultural livelihoods was used to reduce their vulnerability and enhance their resilience in the process of adapting to droughts.

## Discussion

The critical rainfall for crop production in the Zambezi Valley are those occurring between December and February of each year, as this is the growing season of the cereals (Stringer et al. 2009). Although they lack the facilities to measure precipitation, the smallholder farmers in the Zambezi Valley often recognised and remembered the rainfall patterns from previous years, and related them to yield production. They understood that their rainy season starts later in the year (as supported by MSD data spanning the period 1957–2005). Accordingly, the smallholder farmers have adapted the timing of preparation of fields and planting of crops so as to reduce the threat to their crops. Studies conducted in the Zimbabwean districts of Rushinga (Nyakudya & Stroosnijder 2011), Buhera and Chikomba (Mutasa 2010) revealed similar adaptation measures.

Food insecurity is highly prevalent in the Zambezi Valley. This is supported by studies conducted by Zimbabwe Vulnerability Assessment Committee (ZimVAC) (2013) and James (2008). One of the major causes of this situation is drought. The high variability of rainfall makes the area more susceptible to drought. The onset and cessation dates of rain also vary widely. This has caused low production of cereals in the valley because farming systems are mainly rain-fed.

Another important finding of this study was the draining of large amounts of resources from the households during periods of droughts. An estimated 40% of the money used for food procurement in times of drought came from income obtained from distress sales of livestock and household property as well as savings. This strategy is described as an asset depletion response to drought (Enfors & Gordon 2008) and is erosive in nature because it does not build the capacity to effectively deal with the drought situation. Instead it exacerbates the situation of smallholder farmers prior to drought. In some cases, this prevented many of the households from improving their dwellings, investing in agri-business, educating their children, and continuing with their normal income generating activities, some of which have higher potential returns. This is likely to reduce the household drought-coping capacity for future seasons (Kiem & Austin 2013). To make matters worse, the ever increasing price of maize, the plummeting price of livestock and sale of asset holdings to purchase food gave the smallholder farmers very low returns on the investments they made to acquire them (Enfors & Gordon 2008; Kiem & Austin 2013).

Although long-term data about the respondents’ welfare status is lacking, and measurements of the extent of household asset depletion would have been useful for the analysis, this shows that the recurring droughts in the valley sustain what could be viewed as a climate-related poverty trap, whereby smaller welfare improvements only last until the next drought (Carter et al. 2007). Therefore, the numerous strategies that are used to cope with the problems of drought in the Zambezi Valley have failed to work well and are likely to be ineffective in the near future (Chambers 2009; CIGI 2009; IPCC 2007; Nyakudya & Stroosnijder 2011). This was also found in the studies conducted in rural communities of Australia (Edwards & Gray 2009; IPCC 2012; Kiem & Austin 2013). Modern irrigation technologies like drip systems and sprinklers are needed to increase productivity and reduce the impact of drought on food security (Hanjra, Ferede & Gutta 2009).

The strategies of RWH, conservation farming and using drought-tolerant crops and short season cultivars in the farming systems have not been effective enough. Whilst RWH mitigated the risk of intra-seasonal dry spells, and conservation farming enabled improved timing of operations, the two strategies have not helped much in severe droughts associated with El Niño events (Nyakudya & Stroosnijder 2011). This is because during such periods the total rainfall is lower than the water requirements of maize crop, which are about 480 mm for a 125-day cultivar (Nyakudya & Stroosnijder 2011). Therefore, a drought-tolerant cropping system that incorporates sorghum and pearl millet with a minimum water requirement of 300 mm or less was a better option for smallholder farmers in the valley.

## Conclusion

The consecutive drought seasons adversely affected people's food security in the Zambezi Valley. Our findings clearly indicate that smallholder farmers’ dependence on rain-fed agriculture has resulted in high levels of food insecurity. They also illustrate that households quite often deplete their assets during droughts, leading to what seems to be a climate-related poverty trap. Various strategies are used by smallholder farmers to cope with the drought impact. These strategies include crop variety diversification, asset sales for cereal purchases, NGO food aid and gathering wild fruit. The smallholder farmers in the valley have also resorted to a number of measures taken before, during and after a drought. Their activities resemble the disaster management cycle and include RWH practices and conservation and precision farming techniques. However, these strategies are not robust enough to cope with this uncertainty as food insecurity remains high. Therefore, there is need to invest in and adopt irrigation farming throughout the whole year to ensure food security in the valley.
